# The Mediational Role of Desire for Cultural Tightness on Concern With COVID-19 and Perceived Self-Control

**DOI:** 10.3389/fpsyg.2021.713952

**Published:** 2021-09-14

**Authors:** Silvana Mula, Daniela Di Santo, Michele J. Gelfand, Cristina Cabras, Antonio Pierro

**Affiliations:** ^1^Department of Developmental and Social Psychology, Sapienza University of Rome, Rome, Italy; ^2^Department of Psychology, University of Maryland, College Park, MD, United States; ^3^Department of Education, Psychology, Philosophy, University of Cagliari, Cagliari, Italy

**Keywords:** COVID-19, threat, cultural tightness-looseness, desire for tightness, self-control

## Abstract

When ecological threats are more severe or prevalent, societies are more likely to tighten their social norms and punishments. Moreover, when people follow clear and tight rules, they are more prone to regulate their behavior (i.e., self-control) in order to avoid punishment. Therefore, we examined the mediating role of people’s endorsement of cultural tightness (i.e., support and desire) on the relationship between concern with COVID-19 threat and personal self-control. Our hypothesis was tested through a mediation model in two studies with a sample of (*N*=315, 77.1% females, *M*_age_=23.71) university students (Study 1) and with a heterogeneous sample of (*N*=239, 65.7% females, *M*_age_=36.55) participants (Study 2). Empirical support for the proposed model was found in both studies. Implications of this research will be discussed. The main implication is related to the possibility that people’s desire for strong norms to cope with the COVID-19 threat could promote greater self-regulated preventive behavior in order to protect their health.

## Introduction

On March 11th, 2020, WHO defined the spread of coronavirus, or COVID-19, as a worldwide pandemic. COVID-19 is an infectious disease that is currently representing a global health threat. For several months, Italy was one of the countries in the world with the highest number of infected and deaths (Our World in Data, 2021).

Recent studies defined ecological threats, such as pathogens outbreaks, invasions, wars, population density, resource scarcity, or natural disasters, as factors arising from social or natural environment that threaten societies’ existence (Gelfand et al., 2011; Jackson et al., 2019, 2020). Accordingly, the COVID-19 pandemic can be clearly considered an ecological worldwide threat. Previous studies have shown that countries facing territorial threats or spread of diseases feel the need for severe norms and punishment of deviant behavior in order to maintain or restore the social order and group stability (Gelfand et al., 2011; Jackson et al., 2019). In a pandemic, strong social norms are also helpful to prevent and control the spread of the infection. In fact, many countries around the world have strengthened preventive security measures (e.g., social distancing, requirement to wear a mask, mandatory quarantine, and closure of national borders) to contain the transmission of the virus.

Importantly, previous research found that individuals’ desire for cultural tightness would be increased when ecological threats are salient (Jackson et al., 2019). Moreover, given that strong social norms and intolerance for deviant behaviors are needed to maintain social order and stability, cultural tightness has been found to be associated with higher social organization, including higher self-control (Gelfand et al., 2011; Harrington and Gelfand, 2014). Individuals’ self-control encompasses a wide range of responses and abilities, such as better performance regulation, ability to inhibit impulses, exert control over, avoid temptations, and goal-inhibiting impulses (Tangney et al., 2004; Ent et al., 2015; Hagger et al., 2018). Self-control could also be particularly useful for allowing people to follow social regulations. It is now well established from a variety of studies that rule breakers generally exhibit deficits or gaps in self-control (Gottfredson and Hirschi, 1990; Gibbs et al., 1998; Pratt and Cullen, 2000; Gailliot et al., 2012). Given that the restrictions due to the management of the coronavirus pandemic require people to comply with various rules (e.g., stay as much as possible at home, limits on gatherings, and wear masks) in order to limit the spread of the virus and protect its own and others health, it is possible that people who most support these imposed rules may exercise greater self-control and regulation of their behavior. Therefore, based on the previous research, we suggest that concern raised by ecological threat of COVID-19 would increase the extent to which people desire and support tight rules of behavior, and this would, in turn, increase their perceived self-control.

### Ecological Threat, Individuals’ Desire for Cultural Tightness, and Relationship With Self-Control

As mentioned above, when ecological threats are more severe or prevalent, societies are more likely to tighten their social norms and punishments. The term “tightness” was first used by Pelto (1968) to describe the combination of the level of strength of social norms and tolerance for deviant. “Tight” societies, like Singapore and Germany, have strictly defined norms, provide severe punishments on individuals who do not respect these norms, and are described as rigorously formal and disciplined. Conversely, “loose” societies, such as United States and Brazil, have weakly defined norms, are more permissive for norm violating behaviors, and are characterized by a lack of formality and discipline (Pelto, 1968; Gelfand et al., 2011, 2017). In contrast with people in loose societies, those who live in tight ones are generally norm-abiding citizens, have a higher need for stability, and prefer to avoid risks (Gelfand et al., 2006).

An underway pandemic, such as the current one, can create fertile ground for people’s uncertainty and concern. Past research suggests that people generally use a wide range of defensive behaviors and cognitions in response to perceived threats with the aim to restore equanimity and societal order (Gelfand et al., 2011; Jonas et al., 2014).

In this vein, the extent to which people desire and endorse clear and strict rules can play a key role in the context of threat responses. Studies from an evolutionary perspective point out that when nations face collective threats, tight rules, and penalties for deviant behavior may help them to coordinate to survive and reduce chaos to effectively deal with such threats (Gelfand et al., 2011; Roos et al., 2015). Importantly, correlational and experimental evidence by Jackson et al. (2019) showed that societal and ecological threats not only predicted tightness across nations, but also influenced people’s support for cultural tightness.

As previously noted, tight cultures maintain social order and coordination by developing strong norms and intolerance for deviance. To preserve this social cohesion and stability, tight cultures are required to have great social organization, including higher self-control and regulation. In this regard, the previous studies from Gelfand and colleagues (Gelfand et al., 2011; Harrington and Gelfand, 2014; Mu et al., 2015) found that tightness is related to more self-control, while looseness is related to greater impulsivity, reduced cautiousness, and decreased self-regulation and self-control. Results from Harrington and Gelfand (2014) showed that US states that scored higher on tightness also had higher levels of self-control (i.e., drugs and alcohol abuse, high debts). A possible explanation for these findings is that tightly controlled social rules might lead to greater individual self-control. Accordingly, people who live in tight cultures have more impulse control because they must constantly regulate and monitor their behavior to avoid punishment (Gelfand et al., 2011).

## The Present Research

Based on the results obtained from recent research (Jackson et al., 2019), we predict that the COVID-19 pandemic would augment individuals’ endorsement of tight cultural norms. The COVID-19 pandemic is currently a destabilizing threat to societal existence. In the face of the threat, individuals would be more likely to believe that the country in which they live should have strict and clear rules with which people should comply and punish deviants more severely (Gelfand et al., 2011). Indeed, a more restrictive regulatory system could serve to restore social order, contextually helping people better cope with the current threat. Our second prediction is that this desired tightness would, in turn, increase personal self-control (see also Gelfand et al., 2011; Harrington and Gelfand, 2014).

Therefore, we tested a model in which desired tightness would mediate the link between the concern with COVID-19 threat and self-control in individuals. We examined this mediational model in two studies, using different samples (i.e., university students in Study 1 and a more heterogeneous sample in Study 2) and different self-report measures of desired tightness and personal ability to self-control constructs. The studies will be detailed below.

## Study 1

### Method

To estimate the minimum sample size necessary to verify our mediation hypothesis, we used MedPower (Kenny, 2017). The power analysis indicated that at least 163 participants were required for detecting a relatively moderate indirect effect (ab; regression coefficients for a and b paths=0.25, alpha=0.05, and power=0.80). We chose to oversample to increase power.

Three-hundred and fifteen Italian students (243 females, 72 males; *M*_age_=23.71, *SD*_age_=4.74) participated in this study. Their informed consent was appropriately obtained. Data were collected amid the lockdown caused by the coronavirus pandemic in Italy. Participants completed an online questionnaire comprising the set of measures described below. Specifically, measures were presented in the following order: demographic information, that is, gender (subsequently coded as Male=0; Female=1) and age, concern with COVID-19, support for cultural tightness, and self-control.

### Measures

#### Concern With COVID-19

Participants rated their concern about COVID-19 pandemic through an item (i.e., “How concerned are you about the current Coronavirus threat?”) that was responded to on a 7-point Likert scale ranging from “1” (not at all) to “7” (totally).

#### Support for Cultural Tightness Scale

Participants were asked to endorse an ending to nine incomplete statements concerning their support for cultural tightness (Jackson et al., 2019). Each statement (e.g., “My country is currently…”) was responded on a 1–9 scale anchored at “1” (*low* anchor, e.g., “not permissive enough”) and “9” (*high* anchor, e.g., “too permissive”; see also Jackson et al., 2019 for more details on the scale). In the present sample, the reliability of the support for cultural tightness scale was satisfactory (Cronbach’s *α*=0.81).

#### Self-Control

We used the 13-item Brief Self-Control Scale (Tangney et al., 2004) to assess individuals’ self-control. Items were rated on a 5-point Likert scale, anchored from “1” (not at all) to “5” (strongly). The reliability of the Brief Self-Control Scale in this sample was satisfactory (Cronbach’s *α*=0.82).

### Results

Descriptive statistics and bivariate correlations between variables are presented on Table 1. The concern with COVID-19 was positively correlated with the support for tightness. As expected, there was a positive and significant correlation between support for tightness and self-control.

**Table 1:** Bivariate correlations and descriptive statistics (Study 1).

	1	2	3	4	5	*M(SD)*
Gender	–					–
Age	−0.036	–				23.71 (4.74)
Concern with COVID-19	0.292^**^	−0.189^**^	–			5.45 (1.49)
Support for Cultural Tightness	0.219^**^	−0.115^*^	0.265^**^	(0.81)		5.98 (1.06)
Self-control	0.102	0.030	0.083	0.193^**^	(0.82)	3.32 (0.64)

*N*=315. Gender was coded as 0=Male and 1=Female; in bracket (Cronbach’s alpha).

* *p*≤0.05;

** *p*≤0.001.

**Figure 1:**
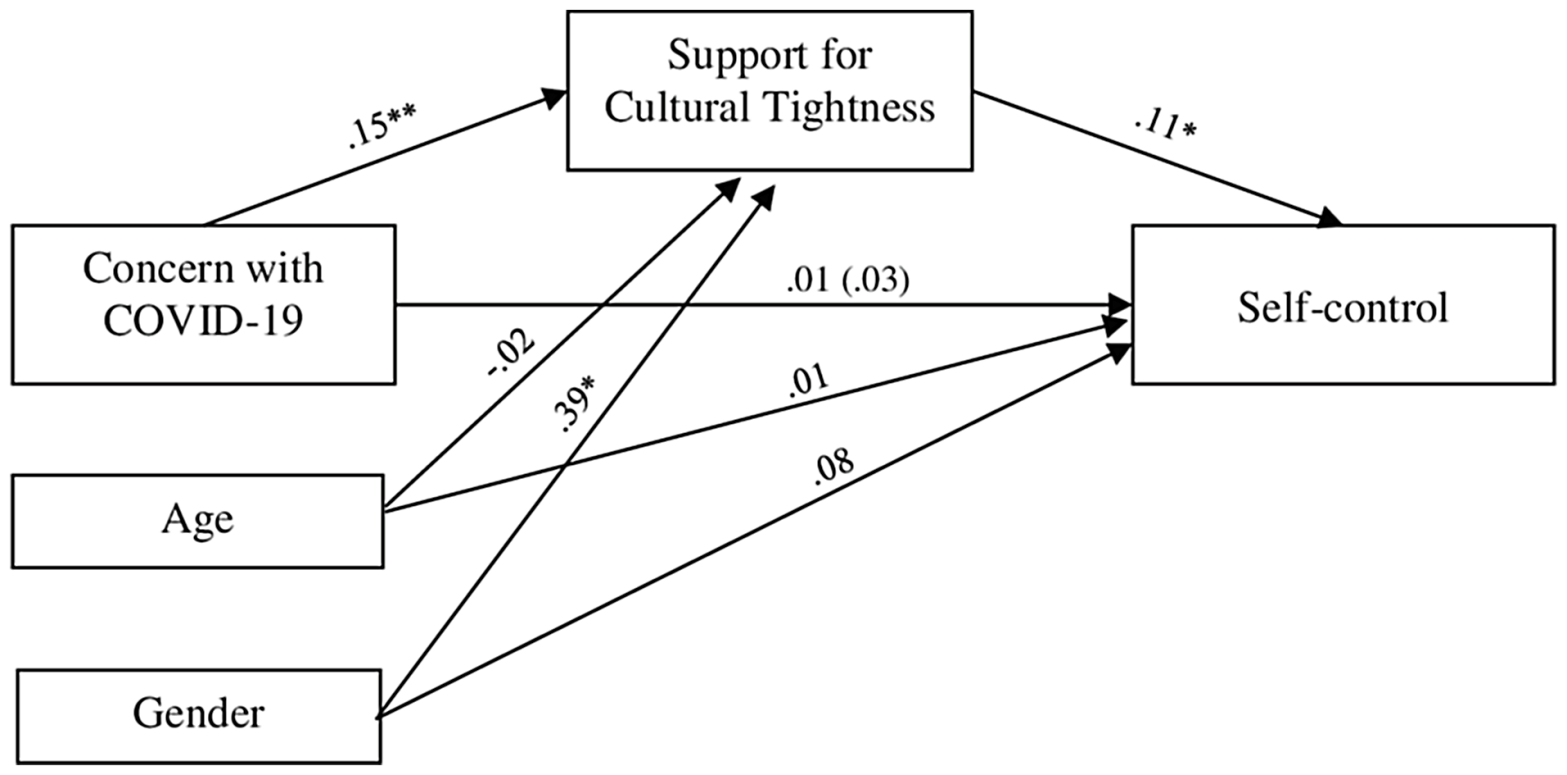
Effects of concern with COVID-19 threat on personal self-control *via* support for cultural tightness. *N*=315. All coefficients are unstandardized. The total effect is inside the parentheses. ^*^p≤0.01; ^**^p≤0.001.

Afterward, we tested the indirect effect of concern with COVID-19 on self-control through people’s support for tightness, and controlling for participants’ age and gender, through the SPSS PROCESS Macro (Hayes, 2017) with 5,000 bootstrap samples and 95% CIs. Results are presented on Figure 1. The support for tightness was significantly predicted by the concern with COVID-19 [*b*=0.15, *SE*=0.04, *t*=3.59, *p*<0.001, 95% CI(0.066, 0.227)]. Self-control was significantly predicted by the support for tightness [*b*=0.11, *SE*=0.04, *t*=3.07, *p*=0.002, 95% CI(0.039, 0.177)]. Most importantly, there was a significant indirect effect of concern with COVID-19 on personal self-control through the support for cultural tightness [indirect effect=0.02, *SE*=0.01, 95% CI(0.004, 0.031)]. Direct effect and total effect were not significant [direct effect=0.01, *SE*=0.03, 95% CI(−0.038, 0.064); total effect=0.03, *SE*=0.03, 95% CI(−0.022, 0.080)]. Age was negatively and not significatively associated with support for cultural tightness [*b*=−0.02, *SE*=0.01, *t*=−1.28, *p*=0.201, 95% CI(−0.040, 0.010]) and positively and not significatively related to self-control [*b*=0.01, *SE*=0.01, *t*=1.03, *p*=0.302, 95% CI(−0.010, 0.023)]. Gender, for its part, was positively and significatively related to support for tightness [*b*=0.39, *SE*=0.14, *t*=2.77, *p*=0.006, 95% CI(0.115, 0.675)] and positively but not significatively linked to self-control [*b*=0.08, *SE*=0.09, *t*=0.95, *p*=0.345, 95% CI(−0.091, 0.260)]. Finally, the entire model was not significant [*F*(3, 311)=1.63, *p*=0.18, *R*^2^=0.02].

Furthermore, we run an alternative model with personal self-control as a mediator and support for cultural tightness as an outcome. No significant indirect effect was found [indirect effect=0.01, *SE*=0.01, 95% CI(−0.014, 0.042)].

### Discussion

The results of the Study 1 showed that people’s support of tightness mediated the relationship between the concern for COVID-19 and personal self-control. Since these results were obtained with the data by self-report measures, they may have biased for the specific measure. In order to address this potential limitation, we conducted the Study 2 using different self-report measures of the same constructs (i.e., desire for cultural tightness and personal self-control), as will be detailed below.

## Study 2

### Method

As well as for Study 1, the power analysis indicated 163 participants as a minimum sample size necessary to detect a relatively moderate indirect effect. Even in this case, we chose to oversample to increase power.

We conducted this study with 239 (157 females, 82 males; *M*_age_=36.55, *SD*_age_=13.59) participants from Italy. The sample was composed of 30% students, 63% workers, and 7% other categories (e.g., unemployed, retired, and housewives). Data were collected outside the lockdown period, but when regulatory restrictions to manage the COVID-19 pandemic (i.e., absolute obligation to wear a mask, limits on gathering, and curfew and targeted lockdown) were still in place in Italy. Participants gave informed consent to participate in this research, then completed an online questionnaire comprising the same measure of concern with COVID-19 used in Study 1, and different measures of desired tightness and self-control, as described below. As in Study 1, participants were also asked to indicate their gender (subsequently coded as Male=0; Female=1) and their age.

### Measures

#### Desire for Tightness

Participants were asked to answer five questions concerning the extent to which they think that the country they currently live in should have the following characteristics right now, on a response scale anchored from “1” to “9”: “1=Have flexible social norms,” “9=Have rigid social norms”; “1=Treat people who don’t conform to norms kindly,” “9=Treat people who don’t conform to norms harshly”; “1=Have fewer rules,” “9=Have more rules”; “1=To be permissive,” “9=To be restrictive; and “1=Be tolerant of those who violate the rules,” “9=Be intransigent with those who violate the rules.” High scores indicated a high desire for tightness (Cronbach’s *α*=0.84).

#### Self-Control

We asked the participants two questions about how often they feel they are successful in maintaining their self-control (i.e., “In general, how often do you succeed in resisting temptations?” and “In general, how often do you succeed in pulling yourself together to pursue a goal you have?”). Each item was rated on a 7-point scale to “1”=never (0% of the time) to “7”=always (100% of the time). The two items constituting this measure of self-control correlated positively and significantly with each other *r*=0.36, *p*<0.001, then we averaged them into a single score of self-control.

### Results

Descriptive statistics and bivariate correlations between variables are presented on Table 2. Confirming the results of Study 1, the concern with COVID-19 was positively correlated with the desire for tightness, and there was a positive and significant correlation between the desire for tightness and self-control.

**Table 2:** Bivariate correlations and descriptive statistics (Study 2).

	1	2	3	4	5	*M(SD)*
Gender	–					–
Age	−0.019	–				36.55 (13.59)
Concern with COVID-19	0.202^*^	0.232^**^	–			5.40 (1.47)
Desire for Tightness	0.089	0.220^**^	0.226^**^	(0.84)		6.34 (1.56)
Self-control	−0.007	−0.027	0.049	0.203^*^	–	5.23 (1.00)

*N*=239. Gender was coded as 0=Male and 1=Female; in bracket (Cronbach’s alpha).

* *p*≤0.01;

** *p*≤0.001.

**Figure 2:**
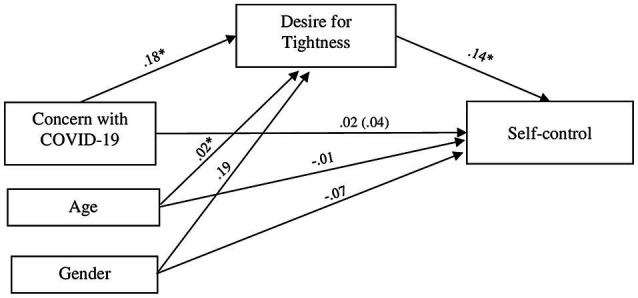
Effects of concern with COVID-19 threat on personal self-control *via* desire for tightness. *N*=239. All coefficients are unstandardized. The total effect is inside the parentheses. ^*^p≤0.01; ^**^p≤0.001.

As in Study 1, we tested the indirect effect of concern with COVID-19 on self-control through desired tightness, controlling for participants’ age and gender, through the SPSS PROCESS Macro (Hayes, 2017) with 5,000 bootstrap samples and 95% CIs. Results are presented on Figure 2. Importantly, the desire for tightness was significantly predicted by the concern with COVID-19 [*b*=0.18, *SE*=0.07, *t*=2.63, *p*=0.009, 95% CI(0.046, 0.321)], and self-control was significantly predicted by the desire for tightness [*b*=0.14, *SE*=0.04, *t*=3.26, *p*=0.001, 95% CI(0.055, 0.222)]. Most importantly, confirming the results of Study 1, there was a significant indirect effect of concern with COVID-19 on personal self-control through the desire for tightness [indirect effect=0.03, *SE*=0.01, 95% CI(0.003, 0.058)]. Direct effect and total effect were not significant [direct effect=0.02, *SE*=0.05, 95% CI(−0.074, 0.108); total effect=0.04, *SE*=0.05, 95% CI(−0.048, 0.134)]. Age was positively and significatively associated with desire for tightness [*b*=0.02, *SE*=0.01, *t*=2.81, *p*=0.005, 95% CI(0.010, 0.035)] and negatively but not significatively linked to self-control [*b*=−0.01, *SE*=0.01, *t*=−1.22, *p*=0.225, 95% CI(−0.016, 0.004)]. Gender was positively and not significatively related to support for tightness [*b*=0.19, *SE*=0.21, *t*=0.90, *p*=0.367, 95% CI(−0.224, 0.603)] and negatively and not significatively associated to self-control [*b*=−0.07, *SE*=0.14, *t*=−0.51, *p*=0.614, 95% CI(−0.340, 0.201)]. Finally, the entire model was not significant [*F*(3, 235)=0.35, *p*=0.79, *R*^2^=0.004].

As for the Study 1, we run an alternative model with personal self-control as a mediator and support for cultural tightness as an outcome. Again, no significant indirect effect was found [indirect effect=0.01, *SE*=0.01, 95% CI(−0.013, 0.043)].

### Discussion

The Study 2 confirmed the mediation model hypothesized and found in Study 1, using different measures compared to Study 1 (except for the concern with COVID-19), and showing again that people’s desire for tightness mediated the relationship between the concern for COVID-19 and personal self-control.

## General Discussion

In our research, we attempted to expand previous research on the effects of ecological threat on cultural tightness (Gelfand et al., 2011; Jackson et al., 2019, 2020) investigating the impact of (concern for) COVID-19 threat—an extremely fearful threat—on people’s desire for tight norms. In two studies, we confirmed previous findings (Gelfand et al., 2011; Jackson et al., 2019) which indicated that threats lead people to strongly endorse the tightness of their country, believing it can contain the threat and restore the order and stability. Furthermore, based on previous research that found greater impulse control in tight countries (Harrington and Gelfand, 2014), we hypothesized that the greater endorsement for strict rules caused by the threat would increase personal self-control. Our findings were in line with our hypotheses in both studies, showing that desired tightness has mediated the relationship between the concern for COVID-19 and self-control.

This result opens a variety of possibilities. As mentioned above, good self-control is linked with better performance, better adjustment, better social functioning, and more optimal emotional responses (Tangney et al., 2004). Importantly, self-control is negatively associated with maladaptive and positively associated with adaptive behavior (De Ridder et al., 2012), including health-related behavior (Hagger et al., 2009; Crescioni et al., 2011). Given the health crisis the world is facing at the moment, this is a relevant potential application. Increases of COVID-19 cases worldwide over time are often linked to the lack of preventive and healthy behaviors in individuals, such as frequently washing hands, wearing a mask, respecting social distance, and avoiding gatherings. However, the pandemic threat, as we have seen, can increase in individuals the desire for strict rules and consequently their (at least perceived) self-control; therefore, people under threat should be more likely to maintain regulated behavior and resist impulses that could be harmful to them and others at this time in history. For example, a recent cross-national study by Nisa et al. (2021) found that the more people perceive a personal risk to suffer economic losses due to the pandemic (i.e., economic threat), the more they support strict health behaviors to contain the virus (wash hands, avoid crowds, socially isolate, support mandatory vaccination, and quarantine). Although our research did not directly investigate adaptive responses, such as health and protective behaviors, we believe our two studies could serve as a starting point and that future research could examine how improved self-control due to a stronger desire for tightness could lead to concrete protective and preventive behaviors. Moreover, although we used common self-control measure (e.g., Malouf et al., 2014; Ent et al., 2015; Hagger et al., 2018), we recommend behavioral measures by following the lines of research which consider self-report and behavioral measures of self-control not interchangeable (e.g., Allom et al., 2016).

Another limitation that we should acknowledge is that data derived from cross-sectional surveys. Thus, our findings may therefore be subject to common method/source biases. We also should recognize that the correlational nature of the data does not allow us to make inferences about the causality of the relationships between concern with COVID-19 threat, desired tightness, and increased personal self-control. Future studies should provide confirmation for our hypothesis implementing longitudinal and experimental designs (i.e., manipulating perceived pandemic threat; see also Jackson et al., 2019; Study 5) to prove evidence about causal path between these variables. We also encourage future researchers to take into account possible confounding variables we did not consider in these two studies. For example, political orientation, experience of threat (e.g., whether participants or someone they know have contracted COVID-19 or how severely COVID-19 affected their country of residence), or sensitivity to threat (e.g., dispositional vulnerability to infectious disease) could be controlled.

In the first study, over than 70% of participants were female, so we tried to address this issue in the Study 2 with a more gender balanced sample. Moreover, especially in online surveys, there could be the possibility that participants respond randomly. We suggest future studies to take this issue into consideration by inserting attention check items, for example.

Another issue to note is that we focused on individual-level of concern and tightness-looseness endorsement. Accordingly, one may argue that the current hypothesis should hold more strongly for predictors that concern the group as a whole. Although we did not examine country-level tightness (which tends to looseness in Italy; *cf.* Gelfand et al., 2011), Jackson and colleagues found no significant differences between tight and loose countries in individual support for tightness caused by ecological threat. Future research combining the study of group-level and individual-level predictors in multi-level designs would be particularly valuable in addressing these issues. We must also recognize that since both studies were conducted in Italy, future research is recommended to test the model in other countries to increase the external validity of these results.

It is also important to note that the association between concern with COVID-19, support and desire for tightness, and self-control may have changed over time since the beginning of the pandemic. People might still be worried about COVID-19, but it is certainly a different concern than when the pandemic started, which was mostly dominated by people’s uncertainty. This varied concern could, consequently, influence people’s endorsement of tightness and their self-control. New investigations are needed considering the ongoing course of the pandemic.

Despite these limitations, the novelty of this research was to examine the impact that a concrete health threat is having on people’s support of strong cultural norms, and how this could potentially translate into adaptive responses of individuals that can help deal with serious threats. Knowing as much as possible the individual and cultural factors that help people, as well as institutions, to better cope with potentials new waves of COVID-19 and future pandemics is crucial. In view of the above, our study could provide deeper insight into the role of the support and desire for tightness and self-control in stemming the negative consequences of the COVID-19 pandemic.

## Data Availability Statement

The raw data supporting the conclusions of this article will be made available by the authors, without undue reservation.

## Ethics Statement

The studies involving human participants were reviewed and approved by the Department of Social and Developmental Psychology, Sapienza University of Rome. The patients/participants provided their written informed consent to participate in this study.

## Author Contributions

All authors substantially contributed to conception and design of the study. SM and DDS made substantial contributions in collecting data and drafting the manuscript. AP, DS, and SM made substantial contributions to analysis and interpretation of data. AP, CC, and MG substantially contributed to revising the manuscript critically for important intellectual content. All authors contributed to the article and approved the submitted version.

## Conflict of Interest

The authors declare that the research was conducted in the absence of any commercial or financial relationships that could be construed as a potential conflict of interest.

## Publisher’s Note

All claims expressed in this article are solely those of the authors and do not necessarily represent those of their affiliated organizations, or those of the publisher, the editors and the reviewers. Any product that may be evaluated in this article, or claim that may be made by its manufacturer, is not guaranteed or endorsed by the publisher.
